# Systematic critical review of previous economic evaluations of smoking cessation during pregnancy

**DOI:** 10.1136/bmjopen-2015-008998

**Published:** 2015-11-13

**Authors:** Matthew Jones, Sarah Lewis, Steve Parrott, Tim Coleman

**Affiliations:** 1 Division of Primary Care, University of Nottingham, Nottingham, UK; 2 Division of Epidemiology and Public Health, University of Nottingham, Nottingham, UK; 3 Department of Health Sciences, University of York, York, UK

**Keywords:** PRIMARY CARE, HEALTH ECONOMICS, STATISTICS & RESEARCH METHODS, SYSTEMATIC REVIEW

## Abstract

**Objective:**

To identify and critically assess previous economic evaluations of smoking cessation interventions delivered during pregnancy.

**Design:**

Qualitative review of studies with primary data collection or hypothetical modelling. Quality assessed using the Quality of Health Economic Studies checklist.

**Data sources:**

Electronic search of 13 databases including Medline, Econlit, Embase, and PubMed, and manual search of the UK's National Institute of Health and Care Excellence guidelines and US Surgeon General.

**Eligibility criteria for selecting studies:**

All study designs considered if they were published in English, evaluated a cessation intervention delivered to pregnant women during pregnancy, and reported any relevant economic evaluation metric (eg, cost per quitter, incremental cost per quality adjusted life year).

**Results:**

18 studies were included. 18 evaluations were conducted alongside clinical trials, four were part of observational studies, five were hypothetical decision-analytic models and one combined modelling with within-trial analysis. Analyses conducted were cost-offset (nine studies), cost-effectiveness (five studies), cost-utility (two studies), and combined cost-effectiveness and cost-utility (two studies). Six studies each were identified as high, fair and poor quality, respectively. All interventions were demonstrated to be cost-effective except motivational interviewing which was dominated by usual care (one study). Areas where the current literature was limited were the robust investigation of uncertainty, including time horizons that included outcomes beyond the end of pregnancy, including major morbidities for the mother and her infant, and incorporating better estimates of postpartum relapse.

**Conclusions:**

There are relatively few high quality economic evaluations of cessation interventions during pregnancy. The majority of the literature suggests that such interventions offer value for money; however, there are methodological issues that require addressing, including investigating uncertainty more robustly, utilising better estimates for postpartum relapse, extending beyond a within-pregnancy time horizon, and including major morbidities for the mother and her infant for within-pregnancy and beyond.

**Strengths and limitations of this study**

- The review implies a broad search strategy of 13 electronic databases, so is likely to have captured most, if not all, of the published literature.
- A quality checklist has been used to systematically identify omissions and limitations of the current literature, and has allowed a qualitative synthesis to allow comparison between interventions in common terms.
- The quality assessment could be considered as subjective, and therefore is possibly influenced by reviewer bias.
- Unpublished trials with published protocols were included, however, other unpublished work was not identified and therefore some relevant evaluations could have been omitted.
- The quality assessment tool is a good judge of studies internal validity but cannot measure external validity, and therefore the tool cannot evaluate the generalisability of the results of included studies.

## Introduction

A major global public health issue continues to be tobacco smoking during pregnancy, with a per annum economic burden conservatively estimated to be £23.5 million in the UK,^1^ and US$110 million in the USA.^2^ Not only is the mother exposed to the long-term risks of smoking,^3^ but has an increased risk of certain pregnancy complications (eg, placenta abruption, ectopic pregnancy),^4^ while also having serious consequences on her offspring.^5–7^ The prevalence of smoking during pregnancy among countries is highly varied, with approximately 39% in Spain,^8^ 23% in Canada,^9^ to 12–14% in the UK, US, Australia and Germany.^10–13^ Suggested explanations for the variation in prevalence are that countries with the higher prevalence also had a greater proportion of mothers with low-household income, low-education levels and low-health literacy levels.^14^ ^15^

Economic evaluation is an important tool for determining which interventions deliver value for money and is an integral part of the decision-making process for new healthcare technologies. However, using the results from poor quality evaluations are likely to lead to misinformed decisions being made and these could have significant negative impacts on health. While economic evaluations of smoking cessation interventions in the non-pregnant population have demonstrated that cessation is cost-effective (offer value for money in terms of effectiveness in relation to cost),^16^ it would appear that similar evidence for within-pregnancy cessation interventions is sparse. A previous review published in 2008 identified only eight studies which involved economic evaluations of cessation interventions delivered to pregnant smokers,^17^ and suggested that such interventions could be considered potentially cost-effective. However, a number of major studies have since been published, so this review could now be considered out of date. The primary aim of this paper was to identify and critically assess economic evaluations of smoking cessation interventions delivered during pregnancy. The secondary aims of this review were to identify any omissions and limitations within previous evaluations, and to determine, which, if any, cessation interventions appeared to be cost-effective.

## Methodology

A previous review conducted by Ruger and Emmons^17^  has already been done on this topic; however, this review could be considered to be out of date as the search was last performed up to July 2003. Furthermore, this review only searched two electronic databases (PubMed and National Health Service Economic Evaluation Database (NHS EED)), and therefore the authors felt that the previous review's search may have missed relevant articles. Therefore, the authors concluded to expand the electronic search and search terms to ensure that a maximum sensitivity search was conducted and that all the relevant literature had been identified.

### Database selection

Thirteen databases were searched: ASSIA, CINAHL, Econlit, Embase, Maternity and Infant Care, Medline, NHS EED, PsycArticles, PsycINFO, PubMed, Tufts Cost-Effectiveness Analysis Registry, Web of Knowledge, and Web of Science. Additionally, the websites of two governmental health guidance bodies, the UK's National Institute for Health and Care Excellence (NICE) and the US Surgeon General, were searched to identify any evaluations published here as part of guideline development.^18^ ^19^ Databases were searched from inception through to August 2014.

### Search terms

The search strategy was developed using terms from a previous review and the Cochrane Pregnancy and Childbirth Group.^17^ ^20^ Search terms and an example search can be found in online supplementary file 1. For the searches of the NICE and US Surgeon General websites, the terms smoking, smoking cessation and pregnancy were used.

### Inclusion criteria

Studies were included if they were in English, reported a formal economic evaluation, with a direct comparison between costs and outcomes, for example, ‘cost per quitter’.

*Population*: Women who had experienced a cessation intervention during pregnancy, and/or their infants/children whose mother had been exposed to a cessation intervention during pregnancy, or hypothetical cohorts modelling cessation during pregnancy and/or after this.

*Interventions*: Any interventions or combination of interventions, both real and hypothetical (an intervention with an assumed quit rate), aimed at encouraging pregnant smokers to quit.

*Comparators*: Any comparator intervention including no intervention and ‘usual care’ (UC).

*Outcomes*: Clinical or economic outcomes considered relevant to the mother and/or child (eg, smoking status at end of pregnancy, low birth weight (LBW) (birth weight <2500 g) births averted, sudden infant deaths (SIDs) averted, and quality adjusted life years (QALYs)).

*Design*: Any type (see table 1 for brief definitions) and design (including within-trial analyses^21^ and decision analytic models (mathematical techniques to synthesise information from multiple sources)^22^) of economic evaluation were considered.

**Table 1:** Brief definition of the different types of economic evaluation

Type of economic evaluation	Definition
Cost-minimisation (CMA)	Interventions are assumed to have equal effectiveness and are ranked in terms of cost (low to high)
Cost-effectiveness (CEA)	Effectiveness of interventions are measured in their natural scale (eg, number of quitters)
Cost-utility (CUA)	Effectiveness of interventions are measured using a generic outcome which embodies health related quality of life which captures a patient's preference (utility) for a particular health state/disease
Cost-benefit (CBA)	Effectiveness of interventions are measured in monetary units
Cost-consequence (CCA)	Costs and consequences of an intervention are reported separately
Cost-offset(COA)	Effectiveness of interventions is measured in healthcare cost savings generated by the intervention

### Exclusion criteria

Exclusion criteria were:

- Studies with no economic analyses
- Studies which focused on the delivery of a smoking service and did not report an outcome that demonstrated the effectiveness of an intervention in terms of health benefits to the mother/infant or reduction in the number of women smoking by the end of pregnancy; examples of irrelevant outcomes include number of general practitioners delivering a cessation intervention, number of women accessing a cessation intervention.

### Identification of papers and data extraction

The lead reviewer screened titles and abstracts of retrieved citations and potentially-relevant texts were retrieved. If a protocol for an ongoing trial was identified, the trial's Principal Investigator was asked to provide economic analysis details. Two reviewers working independently assessed full texts for inclusion, extracted data and applied a quality assessment checklist. If the two reviewers disagreed on data extraction or quality assessment, a third was consulted. A manual search was conducted of references from included studies for other potentially-relevant studies. Papers were then identically screened and reviewed. Data extracted from each study are given in table 2.

**Table 2:** Data extracted from studies

Area of topic	Data extracted
General study background	Author(s)
	Publication year
	Years of study
	Study question
	Funding source
Study design	Study type and design
	Description of intervention
	Description of comparator
	Outcomes measured
	Study assumptions
Evaluation characteristics	Setting (alongside trial vs hypothetical modelling)
	Type of economic evaluation
	Modelling assumptions
	Characteristics of resource estimates (staff time, intervention requirements, hospital use)
	Characteristics of cost estimates (staff cost, itemised costs, total intervention and comparator costs, incremental cost)
	Discounting
	Sensitivity analyses
Study results	Results of evaluation
	Comparison with other evaluations

### Quality assessment

To assess the methodology quality of included studies, the Quality of Health Economic Studies (QHES) checklist was chosen.^23^ The QHES has been demonstrated to be a reliable and valid instrument,^24–26^ and was therefore chosen over other checklists because of its ease of application and the quantitative aspect which would allow comparison across the studies. The QHES contains 16 ‘yes/no’ response questions focusing on the both the methodology of economic evaluations and the broader study, with each question carrying a weighted point score, out of a maximum of 100. The QHES instrument can be found in online supplementary file 2.

When interpreting QHES questions, points were only awarded if the reviewers believed that the most important criteria for the questions were met; if this was the case all points would be awarded. The reviewers did not award fewer points if the study only met some of the question's criteria, the response to each question either being a ‘yes’ (therefore full points) or a ‘no’ (no points). For three individual questions on the QHES (questions five, eight, and 10), the authors specified further criteria to be met in addition to those included within the QHES question. Details of these additional criteria can be found alongside the QHES instrument in online supplementary file 2. Although there is no established, standardised interpretation of the QHES score, the following grouping was adopted based on the work by Spiegel *et al*:^27^ 0–24, extremely poor quality; 25–49, poor quality; 50–74; fair quality; 75–100 high quality.

### Data synthesis

No meta-analysis was specified prior to searches because it was uncertain how studies could be combined; however, the intention was to investigate whether or not this approach would be possible after considering included studies. It was anticipated that the review would adopt a qualitative synthesis, but that a meta-analysis on a subset of data would be investigated if there was potential. The primary objective of the qualitative synthesis would be to discuss the quality of the methods used in identified studies, as determined by the QHES. The results of the assessment from the QHES would be used to demonstrate the strengths and weaknesses of each individual study and of the literature as a whole. To facilitate this QHES scores were allocated to studies as an indicator of overall study quality and qualitatively inspected the components of studies’ scores to investigate which aspects of evaluation quality were commonly absent or poor across studies.

The secondary objectives of the qualitative synthesis were to determine any omissions and limitations of previous evaluations, and to investigate what evidence there was of the cost-effectiveness of within-pregnancy cessation interventions. To allow comparison between the various evaluations, we grouped studies into those who included primary data collection (eg, randomised controlled trials (RCTs)) and those who utilised secondary sources (eg, hypothetical decision analytic models). We adopted this approach as we anticipated that there would be very different assumptions made within the studies, with RCTs likely to be focusing on a short time horizon while decision analytic models a much longer one. Furthermore, decision analytic models often assume background quit rates or intervention/comparator costs which may not be comparable with those collected directly from an RCT.

## Results

**Figure 1:**
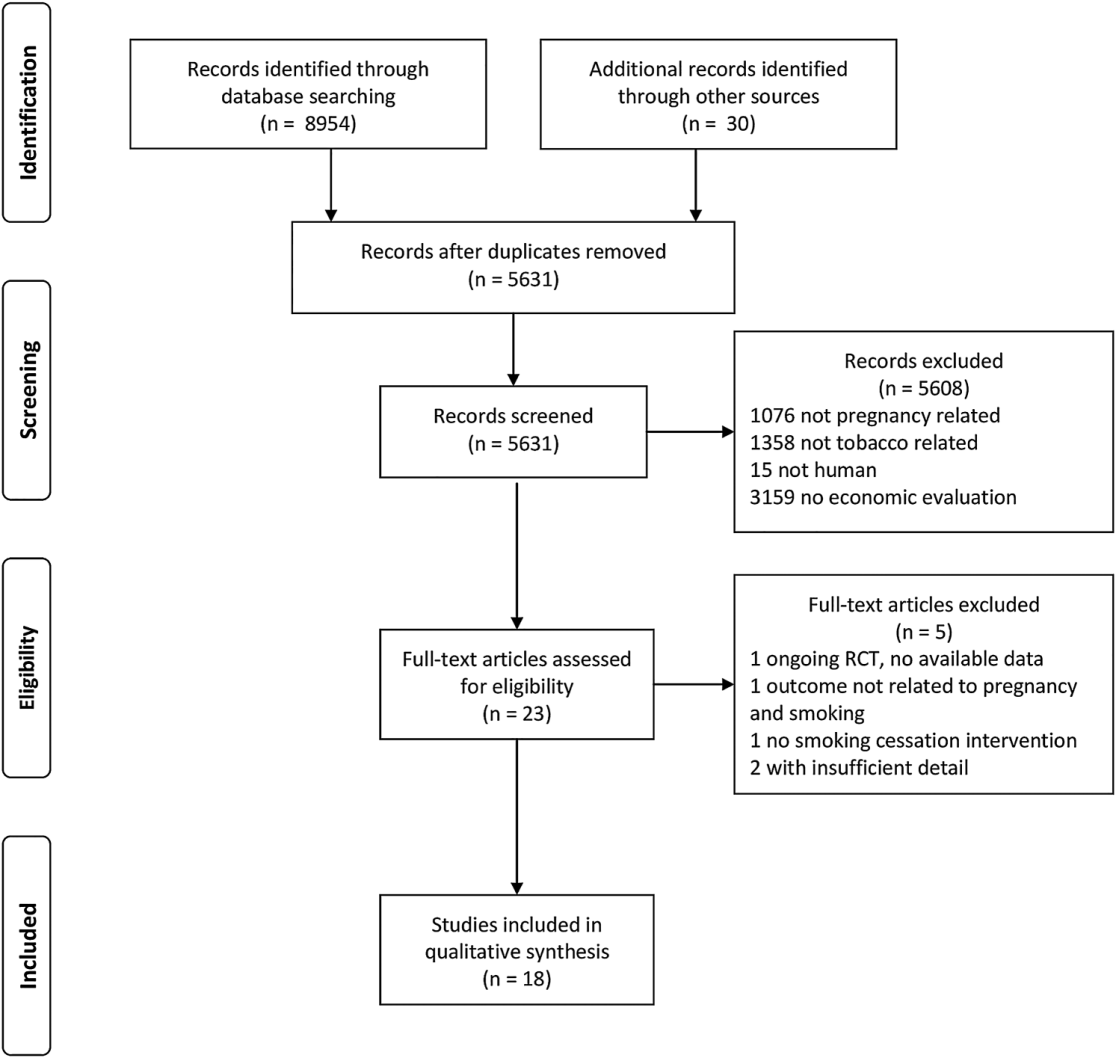
Review PRISMA diagram. RCTs, randomised controlled trials.

The electronic search (conducted 7 August 2014) identified 8954 citations, while the manual searches of the UK's National Institute of Health and Care Excellence (NICE) and US Surgeon General's websites returned a further 30 and zero studies, respectively. Screening identified 23 potential studies, four of which were ongoing randomised control trials (RCTs) with published protocols.^28–31^ Contact with the trials’ Principal Investigators returned the data for three RCTs,^32–35^ while for one, data were unavailable.^30^ Four studies were excluded during data extraction. Two were conference abstracts which reported insufficient detail, and attempts to contact the authors failed.^36^ ^37^ One included no outcomes related to either cessation or pregnancy,^38^ and another did not test a cessation intervention.^39^ The study PRISMA diagram can be found in figure 1. Fifteen studies were published in peer reviewed journals,^32^ ^35^ ^40–52^ two with NICE guidance^53^ ^54^ and one was a unpublished RCTs.^33^ As anticipated, it was decided that a meta-analysis was inappropriate due to the extremely heterogeneous nature of included studies.

### Characteristics of studies

Key characteristics of included studies can be found in online supplementary files 3 and 4. Five studies were conducted in the UK,^32^ ^33^ ^35^ ^53^ ^54^ and the remainder in the USA. There was wide variety in cessation interventions, including: counselling-based (five studies)^40–42^ ^46^ ^50^; self-help materials (two studies);^43^ ^51^ combined self-help materials and counselling (two studies);^48^ ^52^ nicotine replacement therapy (NRT) (one study);^32^ financial incentives (one study)^35^; and physical activity (one study).^33^ Two studies investigated interventions that had previously been described in the literature,^53^ ^54^ while four studies modelled hypothetical interventions.^44^ ^45^ ^47^ ^49^ Comparator interventions among studies with primary data collection were self-help materials (four studies);^41^ ^43^ ^48^ ^52^ brief advice (four studies);^41^ ^48^ ^51^ ^52^ and standard UK National Health Service treatment (see online supplementary file 3 for details) (two studies).^33^ ^35^ The following were used by one study each, placebo patches with behavioural support;^32^ no intervention;^46^ and a cessation programme that was not defined.^42^ For studies without primary data collection, seven used an assumed or spontaneous background quit rate,^40^ ^44^ ^45^ ^49^ ^50^ ^53^ ^55^ while one study used multiple comparators which included low intensity behavioural support, non-conditional incentives and usual care (not defined).^54^

Cost-offset evaluations were used in nine studies,^40^ ^42–45^ ^47^ ^49^ ^50^ ^52^ cost-effectiveness in five,^32^ ^33^ ^41^ ^46^ ^51^ cost-utility in two^53^ ^54^ and two studies used cost-utility and cost-effectiveness.^35^ ^48^ Eight evaluations were conducted within clinical trials,^32^ ^33^ ^41–43^ ^48^ ^51^ ^52^ four were part of observational studies,^40^ ^46^ ^47^ ^50^ five were decision analytic models,^44^ ^45^ ^49^ ^53^ ^54^ and one combined a within-trial analysis with a decision analytic model.^35^ Twelve studies used a healthcare provider perspective (focusing on costs and outcomes directly related to the healthcare provider), while six studies reported a societal perspective (including costs and outcomes directly and indirectly related to the healthcare provider, patient and society as a whole).^32^ ^33^ ^35^ ^48^ ^53^ ^54^

Most evaluations adopted a short time horizon, with 12 studies considering only outcomes during pregnancy or immediately afterwards.^32^ ^33^ ^40–44^ ^46^ ^47^ ^49–51^ Only six studies reported considering outcomes over the mother's lifetime,^35^ ^45^ ^48^ ^52–54^ and two studies incorporated outcomes over the infant's lifetime too.^53^ ^54^ Cost data were predominantly obtained from micro-costing analyses (costing individual component parts separately to generate a total cost for the intervention) collected within clinical trials, with other cost estimates taken from literature sources. Six studies reported discount rates (a rate representing how much individuals discount future health and cost), with rates of 3%,^48^ 3.5%,^35^ ^53^ ^54^ 4%^45^ and 5%.^47^

Measures of smoking cessation were the most frequent primary outcomes (12 studies), while two studies used the number of infants born with LBW (birth weight <2500 g) prevented,^44^ ^45^ one used SIDS (unexplained death within the first year of life) prevented,^47^ and three used QALYs (a life year weighted by the patient's preference for being in a particular health state).^48^ ^53^ ^54^ Secondary outcomes were: LBW infants (six studies),^32^ ^42^ ^43^ ^48^ ^49^ ^52^ premature birth (two studies) (birth occurring before 37 weeks gestation),^43^ ^49^ prenatal death (three studies) (stillbirths and deaths in the first week of life),^32^ ^45^ ^53^ life years (two studies),^48^ ^55^ and QALYs (one study).^35^ When smoking status was used as an outcome in trials, this was biochemically validated in eight studies.^32^ ^33^ ^35^ ^40^ ^46^ ^48^ ^51^ ^52^ Among studies using QALYs, for mothers, one study awarded QALY gains using previously published estimates of QALY gains for quitters,^48^ a second study awarded QALYs on the basis of the mothers smoking behaviour during and after pregnancy,^35^ while a two studies calculated QALYs for the mother taking into account whether the mother smoked postpregnancy and suffered from coronary heart disease, chronic obstructive pulmonary disorder, myocardial infarction, lung cancer or stroke.^53^ ^54^ In addition, one decision analytic model also included QALY losses associated ectopic pregnancy, spontaneous abortion and pre-eclampsia.^54^ For studies including infants, one study used previously published QALY estimates adjusting for the higher mortality rate among children born to smoking women,^53^ while a second awarded QALY losses for birth weight below 2500 g, otitis media and asthma.^54^

Deterministic sensitivity analyses were used to investigate the impact of assumptions made within the study on the results of the economic evaluation in 10 studies;^35^ ^40^ ^44–46^ ^48^ ^49^ ^51–53^ the most frequently varied parameters were intervention effectiveness between high and low quit rates,^40^ ^44^ ^45^ ^48^ ^49^ ^52^ intervention cost between high and low cost,^40^ ^45^ ^46^ ^48^ ^51–53^ and background quit rate between high and low rates.^44^ ^49^ Four studies used robust statistical techniques in probabilistic sensitivity analyses.^32^ ^33^ ^35^ ^54^

### QHES assessment

Table 3 summarises QHES assessment results. Six studies attained a score greater than 75 indicating high quality,^32^ ^33^ ^35^ ^48^ ^49^ ^54^ six were deemed of fair quality,^41–45^ ^53^ and six poor.^40^ ^46^ ^47^ ^50–52^ The median score was 58, with a range from 33 to 87, and an inter-quartile range of 38. Areas where studies seemed to perform poorly were: performing a robust analysis of uncertainty (Q5, four studies), inclusion of all major short-term and long-term maternal and fetal outcomes (Q10, no studies), and incorporation of a time horizon that included the effects within-pregnancy and lifetime for the mother and infant (Q8, one study).

**Table 3:** Results of the QHES assessment

Author	Year	Q1	Q2	Q3	Q4	Q5	Q6	Q7	Q8	Q9	Q10	Q11	Q12	Q13	Q14	Q15	Q16	Total
Ayadi	2006	X	X							X			X			X		35
Cooper	2014	X	X	X	X	X	X	X		X		X	X	X	X	X	X	87
Dornelas	2006	X		X			X	X		X		X	X	X		X	X	67
Ershoff	1983	X					X	X		X		X	X	X		X	X	59
Ershoff	1990	X	X	X			X	X		X		X	X	X		X	X	71
Hueston	1994	X					X	X				X	X	X	X	X	X	57
Mallender	2013	X		X		X	X	X	X	X		X	X	X	X	X		86
Marks	1990	X		X				X		X		X	X		X	X		57
Parker	2007		X					X		X		X			X		X	33
Pollack	2001	X						X				X			X	X	X	36
Ruger	2008	X	X	X	X		X	X		X		X	X	X	X	X	X	78
Shipp	1992	X	X	X			X	X		X		X	X	X	X	X	X	77
Tappin	2015	X	X	X	X	X	X	X		X		X	X	X	X	X	X	87
Taylor	2009	X					X	X		X		X	X	X		X		56
Thorsen	2004	X						X		X					X	X	X	37
Ussher	2015	X	X	X	X	X	X	X		X		X	X	X	X	X	X	87
Windsor	1988	X						X		X		X				X		35
Windsor	1993	X		X						X		X	X			X	X	49
Frequency		17	8	10	4	4	11	16	1	16	0	16	14	11	11	17	13	
Percentage		94%	44%	56%	22%	22%	61%	89%	6%	89%	0%	89%	78%	61%	61%	94%	72%	

X=yes on QHES.

QHES, Quality of Health Economic Studies.

### Findings of studies with primary data collection

Ten studies reported the primary collection of cost and effectiveness data,^32^ ^33^ ^35^ ^41–43^ ^46^ ^48^ ^51^ ^52^ with all except one study identified cessation interventions during pregnancy as being cost-effective.^48^ One UK randomised controlled trial (RCT) reported that the intervention was dominant over usual care (dominance occurs when one intervention costs less and is more effective than another).^33^ Other UK RCTs found the incremental cost per additional quitter was £4926 for NRT,^32^ and £1127 for financial incentives.^35^ One RCT extended the within-trial results to lifetime horizon for the mother using a previously developed model,^56^ and estimated an incremental cost per additional QALY of £482 for financial incentives.^35^ The impact of uncertainty was explored in all three UK RCTs. For NRT, the majority of the bootstrapping iterations laid within the north east quadrant, suggesting that NRT was likely to be more effective but more costly than the comparator intervention consisting of placebo patches and behavioural support.^32^ The probability of financial incentives being cost-effective compared to usual care at £20 000–£30 000 per QALY was 70%,^34^ while for physical activity the probability was approximately 75%.^33^

Among US studies, one RCT reported that using a counselling intervention provided no additional benefit in QALYs and was therefore dominated by usual care.^48^ However, other studies found cost-benefit ratios estimated from 2:1^42^ for self-help materials to 2.8:1^43^ for counselling, though one study found the cost-benefit ratio to be between US$1:17.93 to US$1:45.83 for combined self-help materials and counselling.^52^ Another study found an effectiveness to cost ratio of US$1:84.^46^ The incremental cost per quitter was reported as US$298.76 for a counselling intervention^41^; while one study found that for two different self-help material interventions the incremental cost per quitter was US$50.93 and US$118.83.^51^

To allow comparison between these studies, the incremental cost was inflated to 2014 UK pound sterling prices. UK costs were inflated using the Hospital & Community Health Services Pay and Prices Index,^57^ while US costs were inflated to 2014 prices using the Department of Labor's Consumer Price Index Calculator,^58^ and converted to UK pound sterling using the exchange rate of US$1=GBP0.677173 (correct as of April 2015). In addition to the incremental cost per additional quitter, an incremental cost per additional quality adjusted life year (QALY) was calculated. This was done by assuming a QALY gain of 1.94 which was chosen from previous work, based on the mean age of mothers across the included studies ranging from 24 to 28 years.^59^ ^60^ The results of this analysis can be found in table 4.

**Table 4:** Studies with evaluations informed by primary data collection as grouped by quality as judged by the QHES

Study	Intervention	Comparator	Incremental cost (£)	Incremental quit rate (%)	Incremental cost per additional quitter (£)	Incremental cost per additional QALY (£)
Studies judged high quality on QHES (≥75)						
Cooper *et al*^32^	NRT with behavioural support	Placebo with behavioural support	98.21*	1.8	5456.34*	2812.55*
Tappin *et al*^34^	Financial incentives with standard NHS care†	Standard NHS care†	157.36‡	14.0	1124.00‡	579.38‡
Ussher 2015^33^	Physical activity with standard NHS care†	Standard NHS care†	−35.39	1.3	DOMINANT	DOMINANT
Ruger *et al*^17^	Counselling+self-help materials	Brief advice and self-help materials	304.04	−1.6	DOMINATED	DOMINATED
Studies judged fair quality on QHES (50–74)						
Ershoff *et al*^43^	Self-help materials	Self-help materials	16.58	13.6	121.94	62.86
Dornelas *et al*^41^	Counselling	Brief advice with self-help materials	50.23	18.7	268.62	138.47
Ershoff *et al*^42^	Counselling	Smoking cessation programme (not defined)	149.69	11.6	1290.42	665.17
Studies judged poor quality on QHES (≤49)						
Windsor *et al*^52^	Counselling+self-help materials	Self-help materials	4.99	5.8	86.05	44.35
Windsor *et al*^51^§	Self-help materials	Brief advice	7.12	4.0	178.10	91.80
Windsor *et al*^51^§	Self-help materials	Brief advice	7.12	12.0	59.37	30.60
Parker *et al*^46^	Counselling	No intervention	2357.40	13.4	17 592.55	9068.32

*95% CI Inc cost −£214.48 to £410.92, 95% CI ICER per quitter −£11 915.50 to £22 828.78, 95% CI ICER per QALY −£6142.01 to £11 767.41.

†Standard NHS care involves face-to-face counselling, telephone support, and up to 12 weeks of NRT.

‡95% CI Inc cost £155 to £162, 95% CI ICER per quitter £1107.14 to £1157.14, 95% CI ICER per QALY £570.69 to £596.47.

§Windsor 1988 reports two different self-help material interventions versus brief advice, and thus both interventions have been reported separately.

ICER, Incremental Cost-Effectiveness Ratio; NHS, National Health Service; NRT, nicotine replacement therapy; QALY, quality adjusted life years; QHES, Quality of Health Economic Studies.

### Findings from other included studies

Eight studies used previous literature estimates to inform evaluations, with three being evaluations alongside observational studies with assumed quit rates and intervention costs;^40^ ^47^ ^50^ five studies were modelling-based.^44^ ^45^ ^49^ ^53^ ^54^ Two observational studies found that cessation interventions would generate greater cost savings compared to the cost required to deliver the intervention. Ayadi *et al*^40^ reported that an intervention costing US$24 per person, if applied to the US population, would generate US$ 8 million net saving in healthcare costs, a ratio of approximately 1:333 333. Thorsen *et al*^50^ reported savings of US$137 592 for an intervention costing US$15 366 given to low-income women in the US, a ratio of approximately 1:9. One observational study conducted by Pollack^47^ found that a cessation intervention costing US$45 per person would avert 108 SIDs if given to all pregnant smokers in the US, suggesting that the cessation service would cost US$210 500 per SID averted.

Three modelling studies were also conducted in the USA, and reported favourable cost-saving estimates. Marks *et al*^45^ reported that taking into account the long-term costs averted, the ratio of cost savings to intervention cost was 1:3.26. Hueston *et al*^44^ estimated that cessation interventions were cost-effective if the intervention costed US$80 or less in 1989 prices (US$152.73 in 2014 prices) and achieved a 18% quit rate, while Shipp *et al*^49^ estimated that an intervention would be cost-neutral if the cost of delivering the intervention in 1989 prices (2014 prices) was US$32 (US$61.09) or lower. Using the same exchange rate US$1=GBP0.677173 (correct as of April 2015), the values in UK 2014 prices were £103.42 and £41.37, respectively.

Using a model constructed for informing the National Institute of Health and Care Excellence (NICE) in the UK, Taylor^53^ estimated that rewards (interventions where the participant received a financial or non-financial reward for meeting certain criteria) and ‘other interventions’ (not cognitive behavioural therapies (CBT), financial or pharmacological interventions) were dominant over usual care; however, other cessation interventions had favourable incremental cost-effectiveness ratios (a ratio of the difference in cost over the difference in effectiveness), assessed as £4005 per additional QALY for CBT, £2253 per additional QALY for pharmacotherapies, £1992 per additional QALY for feedback and £2253 per additional QALY for stages of change. In another model constructed for NICE to inform guidance on secondary care interventions, Mallender *et al*^54^ reported that even considering short-term outcomes up to 3 years postintervention, behavioural interventions appeared to be cost-effective with incremental cost-effectiveness ratios of £5445 and £1331 per additional QALY for high and low intensity, while incentives were less cost-effective with incremental cost-effectiveness ratios of £41 088 and £60 409 per additional QALY for conditional and non-conditional incentives. However, the incremental cost-effectiveness ratios decreased as the perspective was increased to include the lifetime for the mother and her infant, and reported that all the interventions modelled achieved a 100% probability of cost-effectiveness by £31 000 per additional QALY in the lifetime analysis.

## Discussion

This review found 18 studies which included economic evaluations of cessation interventions delivered during pregnancy, however, only six of these (33%) were judged as high quality. Seventeen studies identified within-pregnancy interventions as being cost-effective, with only one trial reporting that usual care was better than the experimental intervention.^48^ The current evaluations were generally well described, utilised appropriate health outcomes and drew realistic conclusions based on their results. Conversely, aspects where the analyses were in deficit included consideration of all major and relevant fetal and maternal health outcomes, use of an appropriate time horizon, and controlling for uncertainty using statically robust methods.

A limitation of this review is that the QHES is a subjective instrument. This was highlighted by the need for discussion among reviewers to resolve occasional disagreements about how some QHES items related to studies. However, first, the same issue applies to other checklists and therefore this is likely to have been a problem with any quality checklist utilised. Second, there were occasions where the reviewers felt QHES items were difficult to completely address; hence rewarding partial achievement rather than all or none of the available points may have been more appropriate. For example, for QHES question three it might have been appropriate to score in a graded fashion with points awarded being dependent on the different types of study design (eg, eight points for information from systematic review, seven for information from clinical trial). This could have resulted in the points score calculated for each study better reflecting the overall quality of the methods used, potentially providing a more meaningful comparison. Finally, despite being a good measure of internal validity, the QHES does not measure the external validity. Therefore, this review is unable to capture whether the results of the included studies could be generalised to the population, consequently a meaningful comparison across all the studies may not be possible or appropriate. Nevertheless, the reviewers believe that the use of QHES is appropriate to identify, across studies, those aspects of economic evaluations which might require development. Another consideration is that although the review has included several unpublished studies which we identified from published trial protocols, there may be other unpublished studies which have not been included but are relevant to the review; hence this review may not have included all the potential literature.

This review also has three important strengths. The broad search strategy has allowed the review to identify the majority of the literature published, and it is unlikely that an evaluation has escaped being identified, while also updating the previous review.^17^ Therefore, first, this review is the most comprehensive in this subject to date. Second, the use of the QHES has allowed a systematic identification of the shortcomings in the published evaluations. The important impact of identifying the shortcomings of the current literature is that the review demonstrates that the included studies have several important omissions and analytical limitations which future evaluations would need to remedy for more accurate estimation of the cost-effectiveness of within-pregnancy cessation interventions. Additionally, this is the first review that has conducted a qualitative synthesis on all cessation interventions that have been evaluated as part of clinical trials. This allows the comparison of different within-pregnancy cessation interventions, which is novel in this topic area, and hence permits the decision as to which interventions appear to be the most value for money.

We highlighted several limitations with the economic evaluations in which we identified in the literature. Most studies focused on a within-pregnancy time horizon, with only four studies considering the impacts of smoking during pregnancy on longer term outcomes.^35^ ^48^ ^53^ ^54^ However, it is well-established that smoking is associated with serious morbidities that can occur later in life,^3^ as well as health issues for the infant during its childhood (eg, respiratory disease).^61^ Therefore, to determine the cost-effectiveness of smoking cessation during pregnancy, the time horizon must not only capture within-pregnancy impacts, but also impacts over the lifetime, for both mother and infant. A further issue is that all evaluations omit one or more of the major morbidities which are caused by smoking in pregnancy. Most studies omitted maternal comorbidities associated with smoking and pregnancy, for example, placental abruption, placenta praevia, pre-eclampsia.^4^ These can all lead to severe complications during pregnancy, and in a worst case scenario, death to the infant, the mother or both. However, many studies included some adverse, smoking-related birth outcomes and infant morbidities (eg, IBW, premature birth, stillbirth), but rarely included more than one-condition and did not consider any longer term impacts. Some studies have attempted to capture the healthcare cost savings for adverse birth outcomes avoided from cessation,^40^ ^42–45^ ^47^ ^50^ ^52^ but only one included the impact of LBW and asthma on the health of the child across their lifetime; yet this study excluded premature birth.^54^

Another limitation of the current literature appears to be a general failure across studies to consider the impact of relapse to smoking after pregnancy; only four studies attempted to allow for this, and there was considerable variation in relapse rates applied within these.^35^ ^48^ ^53^ ^54^ Relapse is important since the mother's health risks from smoking increases with relapse, as does the infant's exposure to secondhand smoke.^62^ ^63^ Additionally, recent work suggests that if the mother smokes, an infant is over twice as likely to become an adult smoker,^64^ potentially exposing him or her to the associated lifetime adult health risks. Hence, by not including a rate of relapse to smoking after childbirth, most economic models are overestimating the number of mothers who remain abstinent after pregnancy, potentially overemphasising the benefits of smoking cessation.

One final consideration is the small number of studies which robustly control for uncertainty, with only the four most recently completed incorporating statistically robust techniques.^32^ ^33^ ^35^ ^54^ Controlling for uncertainty appropriately is important since it can demonstrate the level of confidence that the decision resulting from the evaluation is the correct one. While in the past one-way and two-way deterministic sensitivity analyses have been considered appropriate for gauging the impact of uncertainty, it is now deemed better to control for all parameter uncertainty through the use of probabilistic sensitivity analysis.^65^ By not controlling for uncertainty, decisions made on cessation interventions could be incorrect, leading to a cost in benefits forgone. The present literature does not allow a reviewer to determine how confident they are that cessation interventions are cost-effective.

Despite the limitations, included studies suggest that cessation interventions may generally be cost-effective, with only one study out of 18 not supporting that conclusion.^48^ From the within-trial evaluations identified, there is evidence that cessation interventions involving physical activity may offer most value for money because they are dominant (saves money and is more effective), however, this was only based on the results of one study, which also demonstrates that there is a degree of uncertainty in the results.^33^ However, the incremental cost per additional quitter and incremental cost per additional quality adjusted life year (QALY) were relatively low for all other interventions except motivational interviewing, the largest being £17 592.55 per additional quitter (£9068.22 per additional QALY).^46^ This was further supported by the evaluations based on models which either returned very favourable cost-offset ratios for the US-based studies and the incremental cost per additional QALY ratios in UK based models, with one study suggesting that all interventions achieved a 100% probability of cost-effectiveness at a willingness to pay of £31 000 per QALY.^54^ Cessation interventions in non-pregnant populations have often been found to be very cost-effective,^16^ and this review would suggest that cessation interventions within-pregnancy continue to meet this criteria. However, in the four studies that utilised a probabilistic sensitivity analysis, there was evidence of uncertainty which may warrant further investigation, and could impact on the estimated cost-effectiveness of cessation interventions. Therefore, it would seem logical that policymakers should continue to fund cessation interventions for pregnant women as current evidence suggest that they offer value for money; however, there is some uncertainty in the results of which the policymaker might wish to be aware.

## Conclusions

This review demonstrates that although smoking during pregnancy is an important public health issue, there are relatively few high quality economic evaluations demonstrating the cost-effectiveness of cessation interventions, and many of these have methodological shortcomings. Although the majority of included studies suggested that within-pregnancy cessation interventions appeared to be cost-effective, the quality of evidence tended to be poor. To become more comprehensive and to estimate cost-effectiveness more accurately, future economic evaluations of smoking cessation in pregnancy should investigate uncertainty more robustly, use better estimates for the postpartum relapse, extend beyond a within-pregnancy time horizon and include the major morbidities for the mother and her infant for within-pregnancy and beyond.
